# Tumorsphere as an effective *in vitro* platform for screening anti-cancer stem cell drugs

**DOI:** 10.18632/oncotarget.6261

**Published:** 2015-10-31

**Authors:** Che-Hsin Lee, Cheng-Chia Yu, Bing-Yen Wang, Wen-Wei Chang

**Affiliations:** ^1^ Graduate Institute of Basic Medical Science, School of Medicine, China Medical University, Taichung City, Taiwan; ^2^ Department of Microbiology, School of Medicine, China Medical University, Taichung City, Taiwan; ^3^ School of Dentistry, Chung Shan Medical University, Taichung City, Taiwan; ^4^ Department of Dentistry, Chung Shan Medical University Hospital, Taichung City, Taiwan; ^5^ Institute of Oral Sciences, Chung Shan Medical University, Taichung City, Taiwan; ^6^ Institute of Medicine, Chung Shan Medical University, Taichung City, Taiwan; ^7^ Division of Thoracic Surgery, Department of Surgery, ChangHua Christian Hospital, ChangHua County, Taiwan; ^8^ School of Medicine, National Yang-Ming University, Taipei City, Taiwan; ^9^ School of Biomedical Sciences, Chung Shan Medical University, Taichung City, Taiwan; ^10^ Department of Medical Research, Chung Shan Medical University Hospital, Taichung City, Taiwan

**Keywords:** cancer stem cells, tumorsphere, targeting therapy, drug screening

## Abstract

Cancer stem cells (CSCs) are a sub-population of cells within cancer tissues with tumor initiation, drug resistance and metastasis properties. CSCs also have been considered as the main cause of cancer recurrence. Targeting CSCs have been suggested as the key for successful treatment against cancer. Tumorsphere cultivation is based on culturing cancer cells onto ultralow attachment surface in serum-free media under the supplementation with growth factors such as epidermal growth factor and basic fibroblast growth factor. Tumorsphere cultivation is widely used to analyze the self-renewal capability of CSCs and to enrich these cells from bulk cancer cells. This method also provides a reliable platform for screening potential anti-CSC agents. The *in vitro* anti-proliferation activity of potential agents selected from tumorsphere assay is more translatable into *in vivo* anti-tumorigenic activity compared with general monolayer culture. Tumorsphere assay can also measure the outcome of clinical trials for potential anti-cancer agents. In addition, tumorsphere assay may be a promising strategy in the innovation of future cancer therapeutica and may help in the screening of anti-cancer small-molecule chemicals.

## INTRODUCTION

Cancers consist of several varieties of cell types such as cancer cells, stroma cells, endothelial cells, and immune cells; cancers are considered as tissues with heterogeneity [1]. Phenotypic and functional diversity arise among cancer cells. In the past two decades, the identification of cancer stem cells (CSCs) and the power of these cells in the establishment of new tumors during experimental implant in animal hosts [2] enabled CSCs to gain much attention in the field of cancer research. CSCs are a sub-population of cancer cells with properties of tumor initiation. CSC propagate through asymmetric division; similarly, differentiated daughter cells form tumor bulk [3] and normal stem cells differentiate into multiple cell types within a tissue or organ via asymmetric division. CSCs hijack the cellular mechanisms in the maintenance of self-renewal property of normal stem cells to maintain the high tumorigenicity of these cancer cells [4]. CSCs targeting has been considered as the key for successful cancer treatment with the increasing relapse rate of cancers toward current chemo or radiotherapy and the link between CSCs and drug resistance of cancers [5].

## DISCOVERY, CHARACTERISTICS AND ISOLATION METHODS OF CANCER STEM CELLS

The first experimental description of CSCs was demonstrated in a leukemia study in 1997 by John Dick's group [6]. Only a subset of acute myeloid leukemia (AML) cells could transfer AML from patients to non-obese diabetic/severe combined immunodeficient (NOD/SCID) mouse hosts [6]. These AML-initiating cells were found to carry the cell surface markers of CD34^+^CD38^−^ similar to normal haematopoietic stem cells (HSCs) [6], implying that normal HSCs are the cell origin of leukaemic transformation. The identification of AML-CSCs led scientists to search corresponding CSCs in solid tumors, including brain [7], breast [8-11], colon [12], head and neck [13], liver [14], lung [15], ovarian [16], pancreatic [17], and prostate [18], as summarized in Table 1.

**Table 1:** Identified CSC markers in several types of cancers

Cancer Type	CSC markers	Reference
AML	CD34^+^CD38^−^	6
brain	CD133^+^, CD44^+^	7
breast	CD24^−/low^CD44^+^, ALDH^bright^, CD133^+^, CD221^+^	8-11
colon	CD133^+^, CD44^+^, CD24^+^, CD166^+^, Lgr5^+^, ALDH^bright^	12
head and neck	CD133^+^, CD44^+^, ALDH^bright^, SP, GRP78^+^, c-Met^+^	13
liver	CD133^+^, CD90^+^, EpCAM^+^/CD44^+^, CD13^+^, SP	14
lung	CD44^+^, CD133^+^, CD117^+^, CD87^+^, SP, ALDH^bright^	15
ovarian	SP, CD133^+^, CD44^+^, CD24^+^, CD117^+^, EpCAM^+^, ALDH^bright^	16
pancreatic	CD44^+^/CD24^+^/ ESA^+^, CD133^+^, c-Met^+^, ALDH^bright^	17
prostate	CD44^+^CD24^−^, CD44^+^/CD133^+^/α2β1^high^, CD44^+^/CD133^+^/ABCG2^+^/CD24-, PSA^−/low^/ALDH^bright^/CD44+/α2β1+	18

The characteristics of CSCs include tumor initiation, resistance to chemo or radiotherapy, metastasis and involvement of tumor vascularization. The propagation of CSCs to maintain the tumor initiation ability refers to self-renewal of CSCs [3] and signaling pathways in maintenance of self-renewal of normal stem cells are also found to be activated in CSCs, including B lymphoma Mo-MLV insertion region 1 homolog (Bmi1) [19], sex determining region Y-box 2 (Sox2)[20], Wnt/β-catenin [21] and octamer-binding transcription factor 4 (Oct4) [22]. CD24^−^CD44^+^ breast CSCs were noted to be more resistant to radiation than non-CSCs [23]; and these cells not only survived radiation treatment but also induced into the active cell cycle to proliferate [24]. CD133^+^ pancreatic CSCs were demonstrated to be resistant to gemcitabine-induced apoptosis [25], the drug frequently used to treat pancreatic cancer patients. We have demonstrated that silencing of heat shock protein 27 (Hsp27) or treatment of HSP inhibitors potentiated breast CSCs to the suppressive effect of Hsp90 inhibitors [26]. Recent study also reveals that tumor transforming factor (TGF)-β signaling in squamous cell carcinoma CSCs enhanced glutathione metabolism and decreased the efficacy of anti-cancer drugs [27]. The invasive phenotype of cancer cells is driven through the epithelial-mesenchymal transition (EMT) program [28]. Induction of EMT in immortalized human mammary epithelial cells by EMT-related transcriptional factors (twist or snail) or TGF-β1 could increase the expression of CD24^−^CD44^+^[29]. CD24^−^CD44^+^ breast CSCs isolated from primary human breast cancer specimens and expressed markers associated with EMT [29]. The direct regulation of Bmi1 by twist, one of the regulatory transcriptional factors in EMT process, has been demonstrated that these two molecules could cooperate to promote the EMT process and tumor initiation capacity of head and neck squamous carcinoma cells (HNSCC) [30]. We have previously shown that Hsp27 regulated the self-renewal of breast CSCs and controlled their invasive phenotype through downregulation of snail [31]. Wettstein et al. further supported our finding by demonstrating that the inhibition of Hsp27 promoted snail degradation and prevented TGF-β induced EMT [32]. Hermann et al. discovered that CXCR4 expression in CD133^+^ pancreatic CSCs determined the metastatic potential to liver without affecting their tumor initiation capability [33], which presented the concept of metastatic CSCs. Recently, Gao et al. discovered that knockdown of CD44, a marker for CSCs of hepatocellular carcinoma (HCC), diminished the metastatic ability in the experimental mouse model [34]. This work demonstrated the function of CSC marker in the regulation of metastasis. CSCs could also contribute to tumor vascularization. The CD44^+^ ovarian cancer cells have been determined to transdifferentiate into endothelial progenitor cells (EPCs). When CD44+ ovarian cells were cultured with Matrigel, these cells expressed EPC marker CD34 through a vascular endothelial growth factor (VEGF) independent inhibitor of nuclear factor kappa-B kinase β (IKKβ)-dependent mechanism [35]. Wang et al. observed that a subpopulation of CD133^+^ glioma CSCs expressed the marker of vascular endothelial-cadherin (CD144), which displayed characteristics of endothelial progenitors [36]. Blocking the VEGF signaling pathway suppressed the maturation of these tumor endothelial progenitors (CD133^+^CD144^+^) into endothelium; and the inhibition of Notch signaling pathway abolished the transition of glioma CSCs into endothelial progenitors [36]. Vasculogenic mimicry (VM) is another type of tumor vascularization that forms vessel-like channels by tumor cells without the involvement of endothelial cells [37]. Liu et al. demonstrated that CD133^+^ MDA-MB-231 cells, which have been demonstrated as CSCs in Brca1-deficient mouse mammary tumors [38], formed VM structures in triple negative breast cancer tissues [39]. These reports suggested that CSCs may support tumor vascularization through direct transdifferentiation into endothelial cells, EPCs or through the VM mechanism.

CSCs are considered as a small population of cancer cells within a bulk tumor mass. The first and essential step in studying CSCs is to separate them from the entire tumor population. Fluorescence-activated cell sorting (FACS) is the first method to isolate CSCs and is based on the differential expression of cell surface markers by labeling CSCs with fluorescent-conjugated antibodies and isolating by FACS cell sorter. The identification of AML-CSCs or breast CSCs by CD34^+^CD38^−^ [6] or CD24^−^CD44^+^ [8] surface markers, respectively, is the two top known examples. In addition to surface markers, FACS could also be applied to fluorescent-based substrates such as BODIPY aminoacetaldehyde (Aldefluor substrate) for isolation of cells with intracellular ALDH activity (ALDH^bright^ cells); these subpopulation of cancer cells have been identified as CSCs in several types of cancers [40]. Side population (SP) analysis is also a method in applying FACS isolation of CSCs, which is based on the efflux of Hoechst 33342 fluorescent dye [41]. Although SP analysis is considered as an alternative marker to isolate CSCs with no known surface markers, Broadley et al. reported that SP cells were insufficient for CSC phenotype in glioblastoma multiforme [42]. The other fluorescence-based isolation of CSCs is dependent on the cellular proteasome activity. Vlashi et al. first demonstrated that the use of the fluorescence protein ZsGreen fused to the carboxylterminal degron of ornithine decarboxylase to differentiate the intracellular proteasome activity among cells, CSCs of human glioma or breast cancer cells displayed a phenotype of reduced 26S proteasome activity [43]. The reduced 26S proteasome activity of CSCs has been also observed in HNSCCs [44]. Although FACS-based isolation of CSCs seems to be promising, the limitation or disadvantage of CSC isolation by FACS is the availability of fluorescent-conjugated antibodies or substrates as well as the high cost of cell sorter. FACS-based isolation of potential CSCs requires further functional examinations to approve their CSC properties, such as *in vivo* tumorigenicity, *in vitro* drug resistance, cell invasion behavior, or self-renewal capability through tumorsphere assay.

Compared with FACS-based method, isolation of CSCs through tumorsphere cultivation does not require a background knowledge on cell surface markers. Tumorsphere cultivation for CSC isolation was first described by Singh et al. in brain tumors [45], a culture method originally used to isolate neural stem cells [46]. In the study of neural stem cells, the formation of neurospheres was considered as an assay of self-renewal capability according to further examination of the multilineage differentiation ability of these floating spheroid cells [46]. This stem cell cultivation method is based on plating single cell suspension at a proper cell density on ultralow attachment surface with the serum-free culture medium in supplementation with several defined growth factors such as epidermal growth factor (EGF), basic fibroblast growth factor (bFGF) and neural survival factor [47]. Tumorspheres derived from human primary brain tumor specimens expressed markers of neural stem cells (CD133 and nestin) and could further induce multilineage differentiation into neuronal cells or astrocytes. In 2005, Ponti et al. applied the mammosphere cultivation method in *in vitro* propagation of mammary stem/progenitor cells to isolate breast CSCs from primary breast cancer specimens and established human breast cancer cell lines [48]. Mammospheres derived from breast cancer cells expressed CD24^−^CD44^+^ markers and displayed a great tumorigenicity when xenotransplanted into mammary fat pads of NOD/SCID mice [48]. To date, tumorspheres are successfully cultured from varieties of cancers such as colon [49], HNSCC [50], lung [51], pancreatic [52], prostate [53], melanoma [54], ovarian [55] and thyroid [56] cancer. Tumorsphere cultivation is widely accepted as a functional assay of self-renewal property of CSCs [47].

## TUMORSPHERES DISPLAY ALL THE CHARACTERISTICS OF CANCER STEM CELLS

Tumorspheres derived from cancer cells have been proven to display characteristics of CSCs. Dieter et al. applied tumorsphere cultivation to analyze the cellular heterogeneity within colon CSCs [57]. The frequency of sphere-forming cells in the entire human primary colon cancer cells was low but the formed colon tumorspheres displayed a significant tumorigeneicity when xenotransplanted into the kidney capsule of immunodeficient IL2RG^−/−^ mice compared with fresh tumor cells derived from the respective original tumor sample [57]. Coulon et al. also demonstrated that human neuroblastoma cells selected by tumorsphere cultivation displayed increased *in vivo* tumorigenicity in orthotopic microenvironment compared with cells propagated in the presence of 10% serum [58]. In the genetically engineered mouse model of breast cancer, 1000 dissociated cells from 3-week-old tumorspheres derived from tumors of mouse mammary tumor virus (MMTV)-Neu or MMTV-Wnt mice could form tumors when transplanted into the mammary fat pads of Rag^−/−^ mice [59]. Morrison et al. also demonstrated that tumorsphere forming cells in murine ling cancer cell lines were more tumorigenic than adherent cells in the syngeneic host [60]. These reports demonstrate that tumorsphere cells display capability in tumor initiation. CSCs are known to display highly invasive phenotype, which is driven through the EMT program [61]. Lichner et al. found that tumorspheres derived from renal cell carcinoma cells showed elevated expression of mesenchymal markers [62]. Tumorspheres derived from human ovarian cancer cell lines displayed a greater *in vitro* invasive ability and *in vivo* metastasis than their parental counterparts [55]. We have also demonstrated that tumorspheres derived from HNSCC cells displayed EMT signatures such as low expression of epithelial marker E-cadherin and high expression of mesenchymal markers such as vimentin, Slug and zinc finger E-box-binding homeobox 1 (ZEB1) [63]. Nonaka et al. found an elevated invasive ability in tumorspheres derived from RSV-M mouse glioma cells associated with the differential expression of metastatic genes [64]. In addition, tumor cells from metastatic site have been reported to be more easily engrafted in immunocompromised mice than those from the primary site. Lee et al. demonstrated that brain metastases of non-small cell lung cancer showed an increased successful rate in the establishment of patient derived xenografts (PDXs) than primary specimens [65]. Tumorspheres from these PDXs were shown to maintain their brain metastatic feature [65]. These reports illustrate the invasive property of tumorsphere cells. Tumorspheres derived from a poor differentiated human HCC cell line were determined to be resistant to several anti-cancer drugs, which was associated with the elevated expression of ATP-binding cassette sub-family G member 2 [66]. The resistance of doxorubicin was observed in mammospheres derived from MCF7 breast cancer cells and was associated with p62-mediated nuclear factor erythroid 2-related factor 2 activation [67]. These reports establish that tumorpsheres display the capability to resist therapy, which is also one of the features of CSC. The supernatant of HCT116 or HT29 colon cancer cells-derived tumorspheres stimulated the *in vitro* tube formation of EPCs from human umbilical cord blood through secretion of VEGF [68]. Tumor microvessel density was significantly higher in tumors derived from C6 rat glioma tumorspheres compared with cells cultured as monolayer, which was mediated by VEGF and stromal-derive factor-1 secreted by tumorspheres [69]. When melanoma tumorspheres grown on Matrigel matrices, the developed laminin-associated networks were negative with the expression of CD31 and positive with CD144 indicating the feature of VM [70]. These laminin networks within melanoma tumorspheres were diminished by knockdown of nestin [70]. We previously demonstrated that mammospheres derived from human breast cancer cells displayed VM activity when plated on a Matrigel coated surface [71]. The VM activity of breast CSCs was mediated by EGF-induced Hsp27 phosphorylation [71]. These results support that tumorsphere cells could be considered as the enrichment of CSCs according to the capability to contribute to tumor vascularization

## TUMORSPHERES AS A RELIABLE ASSAY IN THE DEVELOPMENT OF ANTI-CANCER AGENTS

### Concepts

The discovery of anti-cancer agents is typically by examining the *in vitro* cytotoxic effect of rapid proliferation cancer cells in 2-dimension (2D) adherent condition. After identifying CSCs, the heterogeneity of tumor cells in response to anti-cancer agents becomes an important issue in drug screening. As described above, drug resistance is one of the main features of CSCs and a mechanism of tumor relapse. Identifying agents with anti-CSC activity has been considered as the key for a successful cancer therapy [3-5]. In addition, phenotypic heterogeneity occurs in the CSC population. For example, the markers of breast CSCs have been described as CD24^−^CD44^+^[8] or ALDH^bright^[10]. CD24^−^CD44^+^ cells without ALDH activity could not form tumors when xenotransplanted into mammary fat pads of NOD/SCID mice [10]. The existence of heterogeneity among CSCs indicates that analyzing the expression of CSC markers in response to potential anti-cancer agents may not a suitable assay to determine their anti-CSC activity. In contrast to 2D monolayer method or analyzing the change of CSC markers, tumorsphere assay is considered as a more reliable platform in the discovery of anti-CSC agents.

### Significance

Tumorsphere assay has been widely accepted ro determine self-renewal capability in studying CSC biology. Examining the *in vitro* anti-self-renewal activity of potential anti-cancer agents by tumorsphere assay could reflect their *in vivo* anti-tumorigenic activity. Morrison et al. used proteomic analysis to compare proteome between MCF7 cells derived from tumorspheres and 2D monolayer culture and results revealed that galectin-3 was overexpressed in MCF7 tumorspheres [60]. N-acetyllactosamine, an inhibitor of galectin-3, both inhibited *in vitro* tumorsphere formation and *in vivo* tumorigenicity of MCF7 cells [60]. Hongisto et al. found that drug sensitivity to 102 anti-cancer compounds between 2D monolayer cultured cells and tumorspheres derived from JIMT1 breast cancer cells was different; when compared the gene expression profiles in different culture methods, tumorspheres derived from Matrigel coated surface showed most closely resembled to xenografted tumors [72]. It strongly suggests that tumorsphere assay is more suitable than 2D monolayer method in screening anti-cancer agents. Kim et al. compared the anti-proliferation activity between doxorubicin and paclitaxel in carcinogen-induced primary murine tumor cells with monolayer or tumorsphere assay [73]. Two chemotherapy drugs displayed a similar anti-proliferation activity in monolayer culture; however, doxorubicin showed a better anti-proliferation activity than paclitaxel in tumorsphere assay [73]. Such differences may result from the different gene expression files between cells derived from 2D monolayer or tumorsphere method as Hongisto et al. observed [72] or the different chemical properties among compounds. Indeed, doxorubicin displayed a greater *in vivo* anti-tumor activity than paclitaxel when tested using tumor cell transplantation mouse model [73]. These results provide evidence to support that tumorsphere assay is a more translatable platform than 2D monolayer method in screening anti-cancer agents.

### Feasibility

Established human cancer cell lines or primary tumor cells isolated from enzymatic digestion of cancer patient biopsy could be used as initial materials for CSC enrichment through tumorsphere cultivation without any background knowledge in CSC-specific markers. The cultivation of tumorspheres could be easily conducted in laboratories with their own cell culture equipment but no FACS cell sorter.

### Methodology/strategy

Ultralow attachment culture surface is required for tumorsphere cultivation, which can be purchased from commercial companies or home-made by coating tissue culture plates/dishes with 1% agar [74], polyhydroxyethylmethacrylate polymer as 12% (w/v) [75], or growth factor reduced Matrigel [72]. Although tumorsphere assay is a relatively low-cost method in screening of anti-cancer agents or in the study of CSC biology, the culture conditions of tumorsphere may influence the interpretation of the results. For example, the initial seeding cell number is important should be carefully distinguished between tumorspheres or cell aggregates. To avoid cell aggregation, filtering cell preparation is suggested with 40 μm cell strainer to obtain single-cell suspension before seeding; cell density should be less than 10 cells/μL [76]. We also recommend the addition of methylcellulose with concentration of 0.5% to 1% in tumorsphere culture medium o prevent cell aggregation. The cultivation condition of tumorsphere may be cancer type-dependent. Although the tumorsphere assay used in most studies is based on serum-free condition under the supplement of EGF and bFGF, several reports also included low concentration of serum. Cao et al. demonstrated that fetal bovine serum and β-mercaptoethanol were essential for the tumorsphere formation of mouse neuroblastoma cells derived from *MYCN* transgenic mice [77]. Matrigel could also be benefit in tumorsphere formation, especially for quiescent CSCs [76]. When counting the number of tumorspheres, the diameter of tumorspheres must be observed. A floating cell clump with a diameter less than 50 μm should not be considered as a tumorsphere. Testing the drug effect is highly suggested by applying a secondary tumorsphere formation with dissociated single-cell suspension from primary tumorspheres. The use of a 100 μm cell strainer to obtain spheres with a diameter large than 50 μm is recommended for the collection of primary tumorspheres. The results of tumorsphere assay could be displayed as the changes of total number or size of formed tumorspheres. The CSC characteristics of formed tumorspheres should be determined before using the tumorsphere assay as the platform in screening anti-CSC agents. Tumorigenicity is the most important feature to be examined. Others include the expression of self-renewal-related genes (i.e. Bmi1, Oct4, Sox2, Nanog, etc.), invasive phenotype, and resistance to conventional chemotherapy drugs or radiation.

### Recent progress/examples

Zhou et al. demonstrated that 8-quinolinol preferentially inhibited the sphere formation capability of MCF7 breast cancer cells and displayed a better therapeutic effect and relapse prevention when combination with paclitaxel in MCF7 xenograft mouse model [78]. By using tumorsphere assay, we have demonstrated that quercetin, a plant flavonoid compound, inhibited the self-renewal capability of HNC-CSCs through the downregulation of stemness genes (Oct4, Nanog, nestin) and mesenchymal markers (vimentin, N-cadherin, Twist) [79]. Quercetin also displayed an anti-CSC activity in breast cancer cells through downregulation of Hsp27 expression [31]. We also discovered that resveratrol (3,4′,5-trihydroxy-trans-stilbene), a polyphenolic compound primarily isolated from red wine, could inhibit tumorsphere formation in the *in vitro* and *in vivo* tumorigenicity of HNC-CSCs through induction of apoptosis of HNC-CSCs [63]. Wen et al. reported a phase II trial of buparlisib, a phosphatidyinositol-3 kinase inhibitor, which has been demonstrated to inhibit the growth of human glioma tumorsphere in recurrent glioblastoma patients. Buparlisib was discovered to be well-tolerated in patients and showed reduction of Akt phosphorylation in 4/6 of evaluable patients [80]. Applying Yamanaka factors to reprogram immortalized mammary epithelial cell line MCF10A, Nishi et al. recently reported a method to establish induced CSC-like cells for *in vitro* screening of potential anti-CSC agents [81]. Withaferin A, an Ayurvedic medicine constituent, displayed an activity in reduction of stemness and self-renewal capability, which determined by alkaline phosphatase activity and tumorsphere formation with minimal effect in cell viability by 2D culture and WST-8 assay [81]. This report demonstrated the advantage of tumorsphere cultivation when compared with the 2D monolayer method in current development of anti-cancer drugs or cancer vaccine. For example, tumorsphere assay is used as one of outcome measurements in a clinical trial to evaluate the effect of mammalian target of rapamycin complex 1/2 (TORC1/2) inhibitor INK128 in treating patients with recurrent glioblastoma (NCT02133183 in Table 2). Treatment of TOCR1/2 inhibitors, BEZ235 or INK128, enhanced the mammosphere formation of triple negative breast cancer cells through activation of Notch signaling pathway [82]. This finding implies that the combination of Notch and TORC1/2 inhibitors should be considered in the clinical trials of TORC1/2 inhibitors for triple negative breast cancer. The involvement of tumorsphere assay in clinical trials is summarized in Table 2.

**Table 2:** Application of tumorsphere assay in clinical trials^1^

Tumor type	Status	Main application	ClinicalTrials.gov identifier
Recurrent Glioblastoma	No specified	Tertiary outcome measures	NCT02133183
Lung cancer Colorectal Cancer Breast Cancer	Phase II	Generation of orthotopic xenograft models that recapitulate the parental tumor behavior	NCT01483001
Prostate Carcinoma	No specified	Primary outcome measures	NCT02425800
Recurrent High-Grade Glioma	No specified	Secondary outcome measures	NCT02101905
Esophageal Squamous Cell Carcinoma	Phase II	Efficacy examination in pre-clinical evaluation	NCT02423811

1 The information of listed clinical trials were obtained from the website of ClinicalTirals.gov.

## APPLICATION OF TUMORSPHERE CULTIVATION IN THE DEVELOPMENT OF CANCER IMMUNOTHERAPY

Recently, cancer immunotherapy is considered to provide great potential in future cancer therapy [83]. However, CSCs have been reported to escape from anti-tumor immunity. Schatton and Frank found that the tumor-associated antigen (TAA) MART-1 was not expressed on the surface of ABCB5^+^ melanoma CSCs [84]. Busse et al. compared the expression of TAAs between tumorspheres and the parental adherent cell lines and found no obvious difference in the expression of TAAs; however, the downregulation of major histocompatibility complex (MHC) molecules was observed in tumorspheres [85]. Wu et al. discovered that breast CSCs were resistant to the cytotoxic killing by autologous or allogenic natural killer (NK) cells which were mediated by microRNA-20a-induced downregulation of MICA and MICB, two ligands for the NK activating receptor NKG2D [86]. Another report demonstrated that CD133^+^ glioma CSCs did not express MHC class I or NK cell activating ligands indicating that these cells were resistant to the surveillances of adaptive and innate immunity [87]. MCF7 cells that survived from NK cells-mediated antibody-dependent cell-mediated cytotoxicity showed CSC phenotypes including CD24^−^CD44^+^ markers and mammosphere formation capability [88]. CSCs from transgenic adenocarcinoma of mouse prostate expressed Tenascin-C to inhibit T cell receptor restricted T cell proliferation by interacting with integrin α5β1 expressed on surface of T cells [89]. Although the immune evasion property of CSCs is being reported, other reports provided evidence that inducing CSC-specific immune responses is possible. Visus et al. using ALDHA1 peptide to generate ALDHAl-specific CD8^+^ T cells and demonstrated that these T cells killed more than 70% of ALDH^bright^ cells in established human carcinoma cell lines [90]. Adoptive transfer of ALDHA1-specific T cells inhibited xenograft tumor growth in immunodeficient mice model [90]. By using tumorspheres as the source of tumor antigen, Xu et al. demonstrated that vaccination of dendritic cells with irradiated glioma tumorspheres increased the survival rate of mice with tumor [91]. Zhu et al. discovered an internalizing human single chain antibody with activity in binding and anti-proliferation effect to brain tumorspheres [92]. Snyder et al. recently demonstrated that the expression of podocalyxin in breast cancer cells is required for the formation of mammospheres; and podpcalyxin antibodies displayed a therapeutic potential to block tumor growth and metastasis *in vivo* [93]. The immune responses in against CSCs can only be studied in immunocompetent host. The identification of CSCs in murine tumor cell lines provides a good model to investigate the tumor immunology and immunotherapy of CSCs. Morrison et al. discovered that tumorspheres derived from HM-LLC murine Lewis lung carcinoma cells displayed CSC properties [60]. We have also shown that Sca-1^+^ 4T1 murine breast cancer cells could formed tumorspheres and are highly tumorigenic when compared with Sca-1^−^ counterparts [94]. The discovery of murine CSCs provides an opportunity to further understand the immunology of CSCs and develop immunotherapy to target CSCs. Recently, Xu et al. demonstrated that the expression of programmed death ligand 1 (PD-L1) was elevated in tumor-propagating cells in a mouse model of lung squamous cell carcinoma, which was induced by biallelic inactivation of Lkb1 and Pten [95]. Such results indicate that the need for testing the application of anti-PD-L1 therapy in breaking down the immune suppression tumor microenvironment. Tumorsphere cultivation also provides an advantage in establishing short-term patient-derived cancer cell lines for the clinical evaluation of dendritic cell-based immunotherapy. Wang et al. conducted a phase I clinical trial of immunotherapy in the treatment of HCC patients with autologous dendritic cells with patient-derived tumorspheres [96]. The efficiency rate for short-term patient-derived HCC tumor cells with tumorsphere cultivation in the clinical trial of Wang et al. was 100%, whereas the successful rate using standard tissue culture techniques was only less than 50% [96].

**Figure 1:**
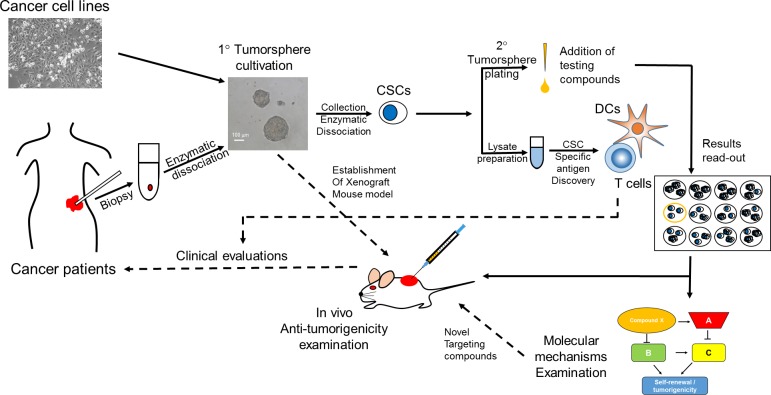
Suggested screening flowchart in the discovery of anti-CSC agents with tumorsphere assay The source of primary tumorspheres can be the established cancer cell lines or primary cancer cells from enzymatic dissociated cancer tissues. Primary tumorspheres could be collected by 100 μm cell strainer and processed to lysate preparation for identification of CSC-specific tumor antigens through dendritic cell presentation and T cell stimulating property. The primary tumorspheres could also be enzymatic dissociated into single cell suspension for secondary tumorsphere formation examination. The potential anti-CSC agents could be tested at this stage. The effects of potential agents will be determined by counting the number and/or the size of formed secondary tumorspheres. The agent with the ability to reduce secondary tumorsphere formation can be selected; the *in vivo* anti-tumorigenic effect of the agent can be determined; and a study on the molecular mechanisms in targeting CSCs can be conducted. Clinical evaluations can be further conducted after obtaining the results of CSC-specific antigen discovery or anti-tumor efficacy of selected anti-CSC agents. CSC, cancer stem cells; DCs, dendritic cells; 1°, primary; 2°, secondary.

Figure 1 shows our view on tumorsphere assay-based discovery of anti-CSC agents. Established human cancer cell lines or primary tumor cells isolated from enzymatic digestion of cancer patient biopsy could be used as the initial materials for CSC enrichment through tumorsphere cultivation (primary tumorspheres). After the dissociation of formed primary tumorspheres, CSCs will be obtained and can be used to examine the anti-self-renewal activity of potential agents by testing the effect in the formation of secondary tumorspheres. Agents with disruption on secondary tumorspheres formation can be selected for further examination of the anti-tumorigenicity activity in immunocompomised mouse model and investigation of the underlying molecular mechanisms by cDNA microarray or protein array-based pathway screening. Moreover, the enriched CSCs from primary tumorspheres can be used as the source of CSC-specific antigens. Through dendritic cell presentation and capability analysis in stimulating T cell proliferation/activation, CSC-specific antigens could be identified. The results from *in vivo* anti-tumor efficacy of the selected anti-CSC agents or the identified CSC-specific antigens could be used as background knowledge for clinical evaluation trials. At this stage, tumorsphere assay could serve as one of the outcome measures.

## CONCLUSIONS AND FUTURE PERSPECTIVES

For the past decades, CSCs have been identified in several types of cancers. This specific subpopulation of cancer cells participates in tumor initiation, resistance to treatment, metastasis, and tumor vascularization; the key for a success cancer therapy is to target CSCs. Cancer cells capable of forming tumorspheres share characteristics with CSCs. Such kind of cultivation method leads scientists to study the biology of CSCs without any background knowledge on CSC markers. Tumorsphere assay could also serve as a reliable platform both in the innovation of anti-CSC agents and the development of CSC-targeting immunothersapy. Several reports have been shown that the screening results from tumorsphere cultivation are more reliable than those from 2D monolayer methods [73, 81]. Given that the immune evasion features of CSCs in some cancer types result from the downregulation of MHC molecules, enhancing the antigen presentation in CSCs must be considered during the development of CSC-targeting immunotherapy. Moreover, understanding the expression of immune checkpoint molecules, such as PD-L1 or indoleamine 2,3-dioxygenase, in tumorspheres of each cancer types can provide useful information when applying immune checkpoint therapeutics against cancers. We believe that tumorsphere assay based-drug discovery of anti-cancer agents will provide more translatable results compared with those obtained from the traditional 2D monolayer culture system.
